# Protective role of taurine in developing offspring affected by maternal alcohol consumption

**DOI:** 10.17179/excli2015-240

**Published:** 2015-05-18

**Authors:** Pilant Ananchaipatana-Auitragoon, Yutthana Ananchaipatana-Auitragoon, Vorasith Siripornpanich, Naiphinich Kotchabhakdi

**Affiliations:** 1 Research Center for Neuroscience, Institute of Molecular Biosciences, Mahidol University, Salaya Campus, Nakhonpathom 73170, Thailand

**Keywords:** maternal alcohol, development, learning, memory, taurine

## Abstract

Maternal alcohol consumption is known to affect offspring growth and development, including growth deficits, physical anomalies, impaired brain functions and behavioral disturbances. Taurine, a sulfur-containing amino acid, is essential during development, and continually found to be protective against neurotoxicity and various tissue damages including those from alcohol exposure. However, it is still unknown whether taurine can exert its protection during development of central nervous system and whether it can reverse alcohol damages on developed brain later in life. This study aims to investigate protective roles of taurine against maternal alcohol consumption on growth and development of offspring. The experimental protocol was conducted using ICR-outbred pregnant mice given 10 % alcohol, with or without maternal taurine supplementation during gestation and lactation. Pregnancy outcomes, offspring mortality and successive bodyweight until adult were monitored. Adult offspring is supplemented taurine to verify its ability to reverse damages on learning and memory through a water maze task performance. Our results demonstrate that offspring of maternal alcohol exposure, together with maternal taurine supplementation show conserved learning and memory, while that of offspring treated taurine later in life are disturbed. Taurine provides neuroprotective effects and preserves learning and memory processes when given together with maternal alcohol consumption, but not shown such effects when given exclusively in offspring.

## Introduction

Alcohol is one of the biggest burden-causing psychoactive substances used worldwide. Alcohol consumption has been an integral part of many cultures for hundreds and thousands of years (McGovern, 2010[24]). Alcohol-related harm is determined by the volume consumed, and is a component cause of more than 200 health problems, notably its dependence, liver cirrhosis and injuries; its uses also contributed to nearly 6 % of global death rate, and 5.1 % of global burdens were attributed to alcohol consumption (WHO, 2014[46]). Mechanisms of harm in an individual occur through toxic effects to tissues and organs, leading to impairment of physical coordination, consciousness, perception, cognition, behaviors, and substance dependence. Alcohol not only is harmful to those drinking it, but it causes problems to those around including socioeconomic burdens, accidence, and household violence.

One of the most alcohol detrimental damages to the world population is through maternal alcohol consumption. The effect of maternal alcohol use not only exerts direct teratogenic impacts on the development of a growing life (For review, see Goodlett et al., 2005[9]), but also results in child maltreatment and neglect (U.S. Department of Health and Human Services, 2014[42]). The most prominent irreversible outcome of alcohol during pregnancy is Fetal Alcohol Syndrome, characterized by retardation of mental development and impaired physical growth especially of the head and face abnormalities. Animal studies also demonstrated that maternal alcohol exposure decreased fetal viability and interfered with the development of the growing embryo (Ghimire et al., 2008[7]); raised an incidence of intrauterine death, tissue malformations and significant growth retardation (Rasmussen and Christensen, 1980[35]). Heavy alcohol intake during pregnancy increases the risk of low birth weight, preterm birth and small gestational age (Patra et al., 2011[33]). Although low-to-moderate prenatal alcohol exposure may not affect birth weights, it can disturb infant behaviors (Chen, 2012[2]).

The detrimental outcomes of maternal alcohol intake most adversely affect the developing brain, causing various intellectual and behavioral deficits. The pronounced outcomes are evident in intellectual deficits consisted of impairment in attention, planning, cognitive flexibility, learning, interpersonal skills and social judgment (Jacobson and Jacobson, 2003[15]). A notable compromised brain function on cognition is an impairment of learning and memory of the affected adult offspring (Hall et al., 1994[11]). Various studies create animal models of Fetal Alcohol Spectrum Disorders (FASD) and reveal affirmative results in neurophysiological alterations associated with fetal and perinatal alcohol exposure. Clements and colleagues (2005[3]) reported that in an animal model of Fetal Alcohol Syndrome, the learning and memory were greatly impaired with evident adverse effects of molecular expression within hippocampus. This coincides with a later animal model study of Fetal Alcohol Syndrome where it was found that neonatal ethanol exposure significantly impaired hippocampus-dependent trace conditioning (Hunt and Barnet, 2014[14]).

Taurine, a conditionally essential, sulfur-containing amino acid, has long been known to provide protective effects and is found abundantly within various vital tissues throughout the body, particularly in the central nervous system. Roles of taurine in the central nervous system are extensive from development to cyto-protection. Taurine acts as a trophic factor during CNS development, in structural integrity of cell membranes, in regulating calcium homeostasis; is an anti-oxidant, an osmolyte, a neuroprotective and a neuromodulating agent. Fetal taurine availability is suggested to be an important determinant of postnatal growth (Hultman et al., 2007[13]). Its deficiency during neonatal period is associated with long-term adverse outcomes on the neurodevelopment and functions (for review see Ripps and Shen, 2012[36]; Verner et al., 2007[43]). Furthermore, The U.S. National Institute of Child Health and Human Development (2007) suggested a standard of practice that taurine is supplemented in formula milk and parenteral nutrition solutions for feeding preterm and low birth weight infants. A recent study also suggested that the most critical period of taurine exposure is in perinatal life, of which effects can contribute to and extend throughout adulthood physiology (Roysommuti and Wyss, 2014[38]). Taurine is also found to be released extensively in aging conditions (Oja and Saransaari, 2013[28]), suggesting its ubiquitous roles throughout life.

More importantly, taurine exerts beneficial roles to guard against cellular disturbances during exposure to toxic substance and can restore impaired functions. It protects cells against oxidative stress (Pan et al., 2010[30]; Yalcinkaya et al., 2009[49]), decreased apoptosis and necrosis (Yalcinkaya et al., 2009[49]). When accompanied by another chelating agent, taurine can increase effectiveness of treatment for lead-poisoning (Gurer et al., 2001[10]). This sulfur-containing amino acid shows protective effects in learning and memory processes. It improves impaired learning and memory of damaged by lead exposure (Hu et al., 2003[12]); reversed the already existed brain damages such as improving learning and memory deficits exhibited in fragile X syndrome (El Idrissi et al., 2009[5]). In addition, taurine also ameliorates age-related cognitive decline of memory acquisition and retention. With chronic taurine supplementation, age-dependent decline in spatial memory acquisition and retention is preserved (El Idrissi et al., 2013[6]).

There are evidence that taurine can preserve tissues and improve the disrupted alcohol damages (Kerai et al., 1998[17]; Olive, 2002[29]; Vohra and Hui, 2000[44]). Importantly, taurine can still exert its protection even when given after alcohol withdrawal (Kerai et al., 1999[16]). As the aforementioned, maternal alcohol consumption is detrimental to the offspring especially intellectual deficits, most notably on cognition, learning and memory (Clement et al., 2005[3]; Hall et al., 1994[11]; Jacobson and Jacobson, 2003[15]; Li et al., 2013[20]). It is also known that taurine can reverse damages caused by various tissue assaults (Gurer et al., 2001[10]; Pan et al., 2010[31]; Rivas-Arancibia et al., 2000[37]; Trachtman et al., 1988[41]; Yalcinkaya et al., 2009[49]), and improve the already damaged nervous tissues and impaired brain functions (El Idrissi et al., 2009[5], 2013[6]; Hu et al., 2003[12]; Kim et al., 2014[19]; Lu et al., 2014[21]; Park et al., 2014[32]; Rivas-Arancibia et al., 2000[37]). It is continually being elucidated that taurine is able to improve alcohol-induced changes (Devi et al., 2009[4]; Kerai et al., 1998[17], 1999[16]; Wu et al., 2013[47]; Yang et al., 2009[50]). And most relevantly, it can restore learning and memory impairment caused by alcohol (Aragon et al., 1992[1]; Vohra and Hui, 2000[44]). These valuable findings lead to a hypothesis that taurine may protect the developing lives from alcohol-induced damages caused by maternal alcohol consumption and preserve the adult offspring's cognition. Accordingly, taurine is the substance of choice to study protective effects against maternal alcohol consumption because

- it interacts with alcohol in liver and brain tissues;
- it is essential during neurodevelopment;
- it protects tissues from various assaults including those from alcohol; and
- it reverses and improves memory impairment including those caused by alcohol.

It can be postulated that during development, taurine can protect tissues from alcohol assaults; thus preserving development and brain functions on learning and memory of growing offspring. We also would like to verify if taurine could reverse disrupted alcohol damages in the developed brain later in life long after intrauterine alcohol exposure had effects on the neurodevelopment in order to further develop corrective measures against fetal alcohol syndrome.

## Materials and Methods

### Study animals and treatments

Eight weeks old adult Mlac:ICR outbred mice (Japanese origin) used in this study were supplied by the National Laboratory Animal Center, Mahidol University, Thailand. Mice weighing 25-30g were mated and pregnant mice at gestational day-GD 4 were housed in pairs (2 pregnant mice per cage) to prevent stress from isolation in a clear plastic cage (19 x 30 x 13cm in size) for general behavioral observations and were maintained in 12-hour light/dark cycles controlled area, with temperatures ranged between 23 °C-25 °C. The pregnant mice were allowed a 3-day environmental adaptation and given water and food ad libitum along with additional liquid diet without alcohol to familiarize the animals with this type of diet, until treatments began. Pregnancy at GD 7 and throughout lactation, mice were randomly assigned to receive different treatments, namely,

- *normal control* - water and food ad libitum without treatments,
- *iso-caloric control* - iso-caloric sucrose in liquid diet, to verify alcohol effects solely independent of its sugar calories as there are suggestive studies on liquid diet and ethanol consumption in relations in weight gain (Mikuska et al., 2013[26]; Smith et al., 2008[40]),
- *alcohol* - 10 % v/v in liquid diet, and
- *taurine - *1 g/kg body weight in drinking water.

The alcohol and iso-caloric sucrose were prepared fresh daily in a nutritionally balanced liquid diet (Ensure, Abbott Laboratories) at a final volume of 25 ml per animal per day. Each treatment group also received water ad libitum and 25 % of daily solid food chow requirements to prevent unnecessary stress from whole liquid diet as mice are rodents. Numbers of each treatment group (n) ranged from 3 to 6 mothers per group.

To investigate whether taurine provides protective effects against alcohol on body growth, development, learning and memory of affected offspring, taurine was given at the dose of 1 g/kg body weight mixed into drinking water. *Maternal taurine supplementation* was given to pregnant mice along with alcohol or iso-caloric pair-fed treatments while *offspring taurine supplementation* was given to post-wean offspring from mothers receiving no taurine, in order to verify if taurine can reverse damages after intrauterine and lactating alcohol exposure. The methods used to ensure that mice took the given taurine was to provide first 10 ml of distilled water mixed with taurine at the beginning of the dark cycle, and then refilled the bottle with plain tap water throughout the rest of the cycles. The same methods were used for other groups, the only difference being that the 10 ml distilled water given initially contained no taurine. Treatments were given at the beginning of the dark cycle when mice were fully awake and alert. Daily liquid diet intake was monitored to measure levels of alcohol consumed by mothers throughout pregnancy and lactation.

### Research group abbreviations

NM = Normal control 

Alc = Maternal alcohol

Suc = Iso-caloric sucrose, pair-fed control

AlcTr = Maternal alcohol + maternal taurine supplementation

SucTr = Iso-caloric sucrose + maternal taurine supplementation, pair-fed control

AlcPWTr = Maternal alcohol + post-wean offspring taurine supplementation

SucPWTr = Iso-caloric sucrose + post-wean offspring taurine supplementation, pair-fed control

### Pregnancy outcomes and offspring body growth

Pregnant mice were weighed every other day to re-assure the pregnancy. The pairs were separated early morning of GD 17 to prepare the mothers of their delivery, each pregnant mouse placed in a private cage (1 mouse per cage). From this day onwards, pregnant mice were checked every few hours for the appearance of pups. As the pups arrived on a particular day, it was defined as postnatal day 0 (P0). Pregnancy outcomes, namely, miscarriage, perinatal death and postnatal mortality, length of gestational days and litter size were recorded. Within 5 hours after delivery, pups were measured for their birth weight and their successive body weight were then monitored weekly until the pups reached 21 days (P21) or weaning, and again at adult age (P56). The pups were weighed with careful handling techniques to prevent unfamiliar odor changes in pups. Laboratory gloves were always worn, hands washed and thin pieces of tissue paper were used to pick up each pup for weight measurement and changed for every pup. Pieces of white clean paper were also laid on top of the scale for each pup and changed consistently. At weaning, offspring were separated from the mother, maintained until adult-P56 in pairs (2 mice per cage) of the same gender in a clear plastic cage sized 19 x 30 x 13 cm in normal housing conditions described above while being given solid food and water ad libitum, with complete alcohol abstinence. Taurine was given to post-wean offspring from maternal alcohol and the iso-caloric pair-fed mothers which received no maternal taurine supplementation. The same method of taurine feeding was performed for offspring taurine supplementation, only that the post-wean offspring were given solid foods and water ad libitum without any liquid diet.

### Learning and memory

Morris water maze, a reliable form of battery of task for rodents, is used to test reference, spatial learning and memory for our study animals, as it is proven to be a robust test with strong correlations with hippocampal synaptic plasticity, variant protocols obtained within 6-day trials (Vorhees and William, 2006[45]). The swimming pool is a delicate balance of motivation provided by stress/reward and a difficult task. Rodents are natural swimmers, thus, making it effective selecting this battery of task performance to test hippocampal-dependent learning and memory (Morris, 2008[27]). The two main advantages of using water maze over other types of mazes are first the mice want to get out, it will start searching for the escape immediately, and second, there are no local cues such as scent trail inside the water as often found using other mazes. Accordingly, questions on performance as a result of traces from other animals prior to them within the maze can simply be disregarded.

In this experiment, the water-maze pool is a round tank filled with water made opaque with nontoxic colloidal white paint (Nippon Odorless). The apparatus (Fill 'n Fun Portable Pool) was 150 cm in diameter, with 30 cm high wall. The pool was placed in the middle of a set-up room surrounded by white walls which were made of white canvas and placed 150 cm away from each side of the pool. Different shapes of cues, namely, stripes, semicircle, and a round clock, were placed on each wall. During tasks, the tank was filled with white opaque water, kept at 20 °C, at a height of 20 cm from the bottom of the pool. Inside the pool was an escape circular non-slippery platform with a diameter of 12 cm; placed 0.5 cm below surface of the water.

The mice were tested in water maze for a period of 6 days after one day pre-training session. Each mouse was performed with a visually guided orientation session, a series of four trials for which the submerged platform had a visible black and white striped cylinder on the top. On the first day, each mouse received a pre-training session consisting of placing the mouse on the platform where it had to stay for at least 15 seconds; followed by a 30-seconds swimming period, and ended by several trials of climbing onto the platform until each subject was able to climb without help. The releasing point differed for each trial (for example, east, west, south, and east if the platform was in the north quadrant) and different sequences of releasing points were used from day to day.

During the task performance, each mouse was subjected to a daily four-trial session. Before the first trial, the mouse was placed for 15 seconds on the platform. Each trial consisted of releasing the mouse into the water facing the outer edge of the pool at one of the quadrants (except the quadrant where the platform was located) and allowing escape to the platform before 90 seconds elapsed. A trial ended when the animal reached the platform, where it was allowed to remain for 15 seconds. The subject that failed to find the platform within the time limit was placed onto the platform and stayed there for 15 seconds before being removed and placed back in a cage for a 15 minutes break interval. At the end of daily sessions, the mice were placed under heat lamp to allow warmth and complete dryness of themselves to prevent stress from cold temperature before being placed back to their home cage. The protocol was adapted from Malleret and colleagues (1999[23]). Performance was recorded using video camera. This allowed for calculations of the escape latency (time required to find the platform, in seconds), percentage of escape per day per group, and learning curves were drawn to illustrate how the subjects learned from the first to the last day of the experiment. 

## Results

### Pregnancy outcomes

All groups carried similar length of gestation (19-21 days) and the litter size was found to range from 12 to 18 pups per litter. There was no maternal death or spontaneous miscarriage from any groups investigated. However, in alcohol treatments, it was found that perinatal death took place (Data not shown). Pups died from both *Alc* and from *AlcTr *groups. 

### Offspring survival rate

**Figure 1:**
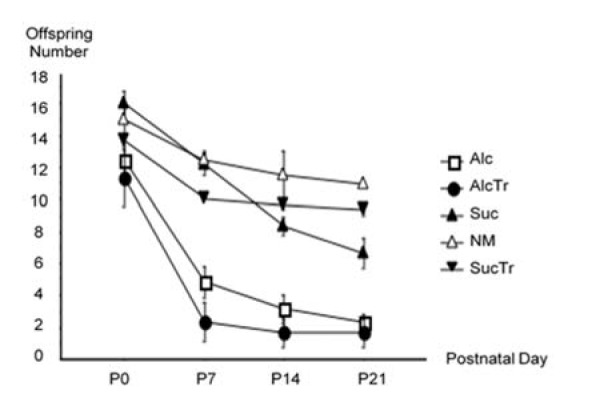
Offspring Survival Rates from Birth (P0) to Weaning (P21), comparison among NM, Suc, Alc, SucTr, and AlcTr groups (n = 3-6 mothers per study group). Data demonstrated as mean ± SEM. ANOVA test revealed that survival rates of the groups examined were significantly different at p < 0.0001 when compared the groups: Alc and Suc, Alc and NM, AlcTr and Suc, AlcTr and NM, AlcTr and SucTr; and at p = 0.0001 when compared the groups of Alc and SucTr. NM = normal control group; Suc = Iso-caloric sucrose, pair-fed control group; Alc = maternal alcohol treated group; SucTr = Iso-caloric sucrose + maternal taurine supplementation, pair-fed control group; AlcTr = Maternal alcohol + maternal taurine supplementation group.

The offspring numbers left per litter differed from controls to treatment groups. Among all groups examined, it was demonstrated that the highest percentage (90 %) of survival was observed of *NM* pups which survived well throughout the experiment. While the lowest (31 %) was pups of *AlcTr*. Interestingly, offspring from maternal alcohol consumption, with or without maternal taurine supplementation, died continuously throughout the research. Our results found only a few pups in both groups left once they reached P21 (Figure 1(Fig. 1)). Maternal alcohol treatment with or without taurine showed significant differences at p = 0.0001 and p < 0.0001 when compared to control groups. Moreover, it was observed that alcohol treated mothers demonstrated both negligence and infancical (killing its own offspring) behavior. When compared among groups at certain ages, it was found at significant level of 5 % that there was a significant difference at p < 0.0001 for P0 and P7, P0 and P14, P0 and P21 and at p = 0.0228 for P7 and P14 and at p = 0.5564 for P7 and P21. The one way ANOVA revealed a significant effect among the treatment groups at p < 0.0001, from birth to weaning at p < 0.0001 and of the interaction between groups investigated and offspring age at p = 0.0076.

### Offspring body weight

**Figure 2:**
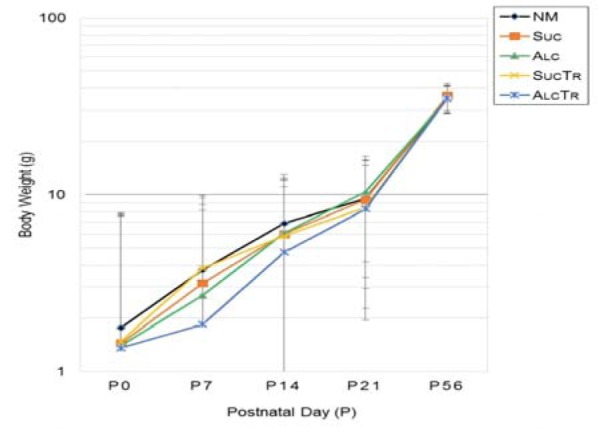
Offspring Successive Body Weight (in gram) from Birth (P0) to Adult (P56), comparison among NM, Suc, Alc, SucTr and AlcTr groups (n = 8-10). Data demonstrated as mean ± SEM. NM = normal control group; Suc = Iso-caloric sucrose, pair-fed control group; Alc = maternal alcohol treated group; SucTr = Iso-caloric sucrose + maternal taurine supplementation, pair-fed control group; AlcTr = Maternal alcohol + maternal taurine supplementation group.

Records of average offspring successive body weight from birth (P0) to adult (P56) revealed that offspring among all groups had similar birth weight. *Alc* and *AlcTr* groups were slightly lower than normal and pair-fed controls without statistical significance. From P0-P21, pattern of weight gain in controls demonstrated a constant, near linear increase, while alcohol treated groups (*Alc* and *AlcTr*) showed a delay at P7, but showed a dramatic catch-up at P21 and entered a comparable weight with all controls at P56, or adult age (Figure 2(Fig. 2)). 

### Learning and memory

**Figure 3:**
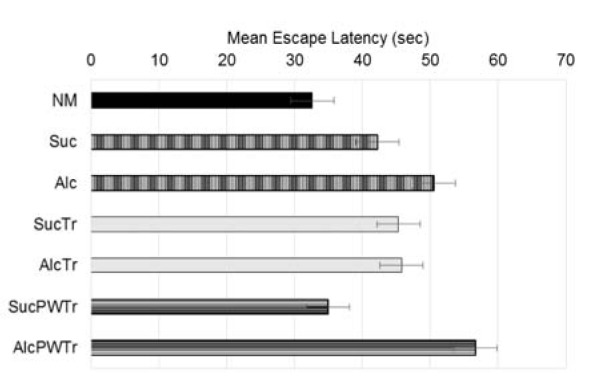
Mean Escape Latency (in second), comparison among NM, Suc, Alc, SucTr, AlcTr, SucPWTr and AlcPWTr groups (n = 6-8). Data demonstrated as mean ± SEM. NM was able to escape within the shortest time (30.13 seconds). When compared among pair-fed groups, it was observed that offspring from AlcTr demonstrated similar time spent compared to pair-fed control (43.87 and 43.35 seconds, for AlcTr and SucTr, respectively). Offspring from Alc showed longer time spent to escape than Suc (48.84 and 40.17 seconds, for Alc and Suc, respectively). And AlcPWTr group also showed longer time spent to escape than pair-fed control (55.23 and 32.51 seconds, for AlcPWTr and SucPWTr, respectively). NM = normal control group; Alc = maternal alcohol treated group; Suc = Iso-caloric sucrose, pair-fed control group; AlcTr = Maternal alcohol + maternal taurine supplementation group; SucTr = Iso-caloric sucrose + maternal taurine supplementation, pair-fed control group; SucPWTr = Iso-caloric sucrose + post-wean offspring taurine supplementation, pair-fed control group; AlcPWTr = Maternal alcohol + post-wean offspring taurine supplementation group.

**Figure 4:**
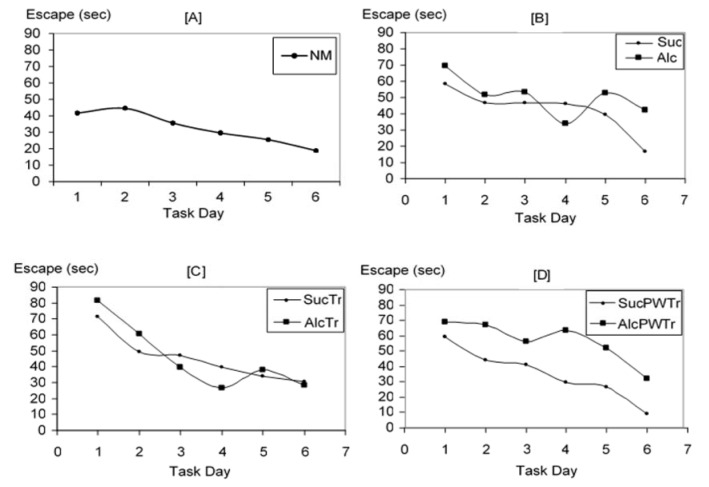
Learning Curves. [A] Illustrates normal learning curve. [B], [C], and [D] Illustrate pair-fed group comparison between *Suc *and* Alc, SucTr *and* AlcTr, SucPWTr *and* AlcPWTr*, respectively (n = 6-8). Data demonstrated as means. NM = normal control group; Suc = Iso-caloric sucrose, pair-fed control group; Alc = maternal alcohol treated group; SucTr = Iso-caloric sucrose + maternal taurine supplementation, pair-fed control group; AlcTr = Maternal alcohol + maternal taurine supplementation group; SucPWTr = Iso-caloric sucrose + post-wean offspring taurine supplementation, pair-fed control group; AlcPWTr = Maternal alcohol + post-wean offspring taurine supplementation group.

We collected the first data on escape latency: the time at which the study animals spent in finding the hidden platform, from day 1 to day 6 of the experiment. Means of this escape latency were then calculated as shown in Figure 3(Fig. 3). From day 1 to day 6 of the experiment, it was found that offspring from *NM *learned the water maze task well and was able to escape with lesser times as the experiment proceeded. The same pattern of learning was also observed in pair-fed control groups. Similarly, the offspring from *AlcTr* demonstrated the tendency to escape requiring lesser time as the experiment proceeded as compared to controls: *NM*, *Suc*, *SucTr *and *SucPWTr*. In contrast, both *Alc* and *AlcPWTr* group did not show a reduction in escape latency as compared to other groups. From these data, learning curves were drawn to illustrate how offspring from each group learned hippocampal-dependent water maze task. Learning curves were plotted from day 1 to day 6 of the experiment, illustrated in Figure 4(Fig. 4). Normal learning curve as observed from *NM* group demonstrates a progressive decline in escape times as the experiment proceeded (Figure 4 [A](Fig. 4)). When compared between treatment and pair-fed control groups, it was similarly observed that mice from *Suc* required lesser time to escape to the hidden platform as the experiment proceeded. On the other hand, *Alc* offspring were lost in the water maze from time to time and did not perform the task well. This data suggested impairment in the learning and memory processes (Figure 4 [B](Fig. 4)). On the contrary, offspring from *AlcTr* group demonstrated a decline in escape latency, requiring lesser time as the experiment proceeded, which is comparable to its pair-fed counterpart *SucTr* on each trial day, indicating a conserved learning and memory processes (Figure 4 [C](Fig. 4)). Moreover, *AlcTr* spent even lesser time to find the platform when the experiment ended on day 6, similar to its pair fed control (28.50 and 30.33 seconds, for *AlcTr* and *SucTr*, respectively, data not shown). In contrast, *AlcPWTr* group spent much longer time in finding the platform than its pair-fed counterpart throughout day 1 to day 6 and depicted an erratic pattern, suggestive of a non-conserved learning and memory (Figure 4 [D](Fig. 4)).

**Figure 5:**
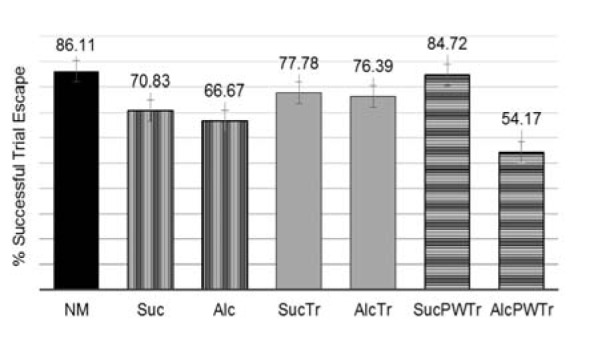
Mean Percentage of Overall Successful Trial Escape, comparison among NM, Suc, Alc, SucTr, AlcTr, SucPWTr and AlcPWTr, groups (n = 6-8). Data demonstrated as mean ± SEM. When analyzed using ANOVA analysis per trials, there was a level of significant difference among trials at p = 0.0001. When analyzed the percentage of escapes among all groups investigated, it was found that they were significantly different at p = 0.0001. NM = normal control group; Suc = Iso-caloric sucrose, pair-fed control group; Alc = maternal alcohol treated group; SucTr = Iso-caloric sucrose + maternal taurine supplementation, pair-fed control group; AlcTr = Maternal alcohol + maternal taurine supplementation group; SucPWTr = Iso-caloric sucrose + post-wean offspring taurine supplementation, pair-fed control group; AlcPWTr = Maternal alcohol + post-wean offspring taurine supplementation group.

Finally we also calculated the percentage of total successful escapes to the platform during task performance. Mean of the percentage is shown in Figure 5(Fig. 5). We found that *NM* showed the highest percentage of escape in this water maze task. Offspring from *Alc* group showed lower percentage in the ability to escape from the water (66.67 %), compared to *Suc* (70.83 %). Offspring from *AlcTr* showed comparable percentage of escape to its pair-fed counterpart (*SucTr* group). In contrast, taurine supplementation given to post-wean offspring did not seem to preserve the learning process as the group was demonstrated to have much lower escape (54.17 %) compared to its pair-fed (84.72 %).

## Discussion

It is clearly evident that maternal alcohol consumption offers no benefits to the growing life. Our current study demonstrates that maternal alcohol exposure at 10 % of liquid diet throughout pregnancy and lactation resulted in perinatal death and infancidal behavior of the mother. Supplementation of taurine supplementation does not protect against perinatal death. Our study also depicts detrimental effects of maternal alcohol consumption on body growth and survival of the offspring. Again, taurine does not protect against alcohol on body weight or survival rates of the affected offspring.

Interestingly, birth weights among all study groups are similar and the offspring is able to catch up in growth approaching weaning age. It is observable that the underdevelopment, as evident in delayed weight gain, is more pronounced after birth, at P7, and not afterwards despite being within lactation, thus still exposed to alcohol. This observation suggests a possibility of other factors apart from direct alcohol teratogenic effects on the offspring intrauterine and postnatal growth. One postulation is that during P0-P7, which is equivalent to the third trimester in humans, there is a rapid growth that alcohol has effects on and results in delayed weight gain. Another potential mechanism is alcohol effects on maternal behaviors, deviating and preventing it from fostering and feeding the offspring. Previous collective information on substance abuse and child maltreatment suggest that alcohol use can impair both physical and mental capabilities for parenting functions (U.S. Department of Health and Human Services, 2014[42]). Our study finds that not only alcohol treated mothers leave their litter unattended and unfed, but it also demonstrates infancidal behavior, killing its own offspring. However, the offspring who survives through the critical period is able to take care of itself; seeks feedings; and continues to grow. This postulation also explains our current finding that taurine does not protect offspring growth and survival. Because alcohol may not exert its effects directly on offspring, that taurine does not have its interaction and protection on.

Our current research employs taurine as a potential protective substance to preserve the learning and memory impairment affected by alcohol. Maternal alcohol consumption is detrimental to learning and memory processes of the affected offspring. Our results showed that alcohol affected offspring elucidate deviated patterns of hippocampal-dependent performance, which are in coherence with previous findings of deficient maze performance (Hall et al., 1994[11]). A molecular study revealed that alcohol-fed animals showed lowered hippocampal c-Fos expression (Clement et al., 2005[3]). Another study reported that alcohol reduced neurogenesis and synaptogenesis (Gohlke et al., 2005[8]). These findings might explain the deficits seen in our study. In contrast, taurine continues to elicit neuro-protection in our results. It preserves task performance of the alcohol affected adult offspring. This information is similar to previous findings on improvement of impaired memory caused by alcohol (Vohra and Hui, 2000[44]) and an interaction of alcohol and taurine in their effects on an open-field loco-motor activity (Aragon et al., 1992[1]).

Improvement in learning and memory of affected adult offspring is observed when taurine is given together with alcohol exposure, but not when given later in life. It is suggestive, as is evident that alcohol and taurine do interact with each other within the nervous system (Olive, 2002[29]). Mechanisms of protection by taurine can be extensive. One possible protection may be exerted through maintaining neural amino acids. It is previously found that taurine supplementation can delay sulfur-containing amino acids metabolisms caused by alcohol (Yang et al., 2009[50]). Thus potentiation of these amino acids availability within the assaulted developing brain may explain its protective effect. Because taurine is found to be predominantly essential during neuro-development and alcohol is found to reduce transport of taurine during this vital developmental neurogenesis (Lui et al., 2014[22]).

The second possible mechanism is the protective effect of taurine in developing neural tissues from alcohol assaults, possibly due to its ability to reduce glutamate-induced excitotoxicity (Wu and Prentice, 2010[48]) and apoptosis (Menzie et al., 2013[25]), particularly in developing hippocampus (Oja and Saransaari, 2013[28]). Preservation of hippocampal-dependent learning and memory process of alcohol by taurine supplementation can be explained through preventing hippocampal excitotoxicity and reducing cell death during critical developmental period. Taurine is found to positively modulate GABAergic interneurons via glycine receptors within the neocortex (Qian et al., 2014[34]; Sava et al., 2014[39]). Recently, tonic GABAergic activity is suggested to be critically involved in physiological and pathological processes during the establishment of functional GABAergic synaptic connections in postnatal brain development (Kilb et al., 2013[18]). Thus making it highly likely that taurine inhibits adverse effects of maternal alcohol within the developing brain through activation of GABAergic activity. Additionally, taurine ubiquitous roles within the body can all potentially provide synergistic effects in protecting the developing brain from alcohol assaults. These include being cerebral osmoprotective (Trachtman et al., 1988[41]), and being protective against lipid peroxidation in the brain (Rivas-Arancibia et al., 2000[37]). Moreover, when being given alongside each other, taurine can help enhance alcohol metabolism and detoxification (Devi et al., 2009[4]; Wu et al., 2013[47]), thus minimizing the chance of reaching the intrauterine developing tissues.

As taurine given alone later in life of the affected offspring does not show conserved learning. It suggests that such brain damages have occurred in early developmental stage. Therefore when taurine is given from weaning onwards, the learning deficits are irreversible.

In conclusion, taurine is found to preserve learning and memory of the affected offspring when given early in life to the mother throughout lactation and provides a promising therapeutic remedy for brain malfunctions caused by maternal alcohol consumption. In contrast, taurine supplementation cannot ameliorate underdevelopment caused by maternal alcohol exposure or provide its protective effects on learning and memory of the affected adult offspring when given later after the development. Further investigations into behavioral studies of mothers consuming alcohol in relations to the effects of negligence on body and brain development, infancidal behaviors, as well as alcohol and taurine interactions upon brain development and intellectual correlates could prove invaluable.

## Notes

Pilant Ananchaipatana-Auitragoon and Naiphinich Kotchabhakdi (Research Center for Neuroscience, Institute of Molecular Biosciences, Mahidol University, Salaya Campus, Nakhonpathom 73170, Thailand; Tel: 6681-483-6066, Fax: 662-889-2155, naiphinich@gmail.com) contributed equally as corresponding authors.

## Acknowledgement

This research is supported by Mahidol University Research Grant.

## Conflict of interest

The authors declare that we have no conflict of interest in this research.
